# Clinical Features, MRI Findings, Treatment, and Outcomes in Dogs with Haemorrhagic Myelopathy Secondary to Steroid-Responsive Meningitis-Arteritis: Nine Cases (2017–2024)

**DOI:** 10.3390/vetsci12050476

**Published:** 2025-05-15

**Authors:** Giuseppe Vitello, Beatrice Enrica Carletti, Sergio A. Gomes, Luca Motta, Alessia Colverde, Andrea Holmes, Massimo Mariscoli

**Affiliations:** 1 Paragon Veterinary Referrals, Part of Linnaeus, Paragon Business Village Paragon Way, Red Hall Cres, Wakefield WF1 2DF, UK; andrea.c.holmes@gmail.com (A.H.); massimo.mariscoli@paragonreferrals.co.uk (M.M.); 2 Dick White Referrals, Part of Linnaeus, Station Farm, London Road, Six Mile Bottom, Cambridgeshire CB8 0UH, UK; beatrice.carletti@dwr.co.uk; 3 Dovecote Veterinary Hospital, Castle Donington, Derby DE74 2LJ, UK; 4 Nortwest Veterinary Specialist, Part of Linnaeus, Runcorn WA7 3FW, UK; luca.motta@nwspecialists.com; 5 Wear Referrals, Part of Linnaeus, Bradbury, County Durham TS21 2ES, UK; alessia.colverde@wear-referrals.co.uk

**Keywords:** steroid-responsive meningitis-arteritis, dog, haemorrhagic myelopathy, diagnostic imaging

## Abstract

This retrospective multicentre study investigated haemorrhagic myelopathy as a rare complication of steroid-responsive meningitis-arteritis (SRMA) in nine dogs. The affected dogs exhibited varied neurological deficits, including cervical hyperesthesia, generalised stiffness, ambulatory tetraparesis, and, in the most severe cases, paraplegia without nociception. MRI findings primarily localised haemorrhagic lesions to the thoracolumbar (T3-L3) region, with intradural–extramedullary haemorrhages being the most common type. Most cases responded favourably to immunosuppressive therapy with prednisolone, either alone or in combination with cytarabine. Surgical intervention, performed in a case of compressive extradural haemorrhage, led to a successful recovery of ambulation. Two cases presented or developed paraplegia without nociception, despite immunosuppression. These findings emphasise the importance of advanced imaging and timely therapeutic interventions in addressing atypical and severe manifestations of SRMA.

## Simple Summary

Steroid-responsive meningitis-arteritis (SRMA) is an immune-mediated inflammatory disease in dogs. Rarely, it can progress to haemorrhagic myelopathy due to systemic vasculitis, a complication that remains poorly documented. This study aimed to investigate the clinical presentation, MRI findings, treatment, and outcomes in dogs diagnosed with haemorrhagic myelopathy secondary to SRMA.

## 1. Introduction

Steroid-responsive meningitis-arteritis (SRMA) is an immune-mediated disease characterised by inflammation of the leptomeninges and associated arteries [1,2,3], and with a prevalence of 1.6–2% within cases presented to neurological departments [4,5]. SRMA can affect any dog breed [1,2,3], but it has been reported affecting more frequently Boxers, Beagles, Bernese Mountain Dogs, Border Collies, English Springer Spaniels, Jack Russell Terriers, Nova Scotia Duck Tolling Retrievers, Weimaraners, and Whippets [1,2,3]. Dogs with SRMA typically present at a young age, between 6 and 18 months [1,2,3]. However, exceptions have been documented, with sporadic cases aged 3 months or younger and as late as 9 years of age [6,7,8]. Although most of the veterinary literature suggests that SRMA affects both sexes equally [6,7,9,10,11], a recent study indicate a possible predisposition in male dogs [12]. Although the exact aetiology of SRMA remains unclear [1,2], it is thought to be an immune-mediated disease due to the rapid resolution of the clinical symptoms after glucocorticoids and/or other immunosuppressive drugs. It was hypothesised that an antigenic stimulus may trigger the disease; however, no infectious (viral, bacterial, or parasitic agents), neoplastic, or non-infectious environmental toxins have been demonstrated as a definitive trigger for SRMA [1,6,7,11,13,14,15,16,17,18,19,20,21]. Two clinical forms of SRMA have been recognised, the typical acute form and the chronic form also defined as atypical [1,6,13]. The acute form is clinically characterised by hyperesthesia along the vertebral column and particularly on the neck, a stiff gait, pyrexia, and increased serum C-reactive protein (CRP). The changes in the cerebrospinal fluid (CSF) are usually not pathognomonic, but a marked neutrophilic pleocytosis, increased protein concentration, and presence of a variable number of red blood cells secondary to leakage from meningeal vessels are likely indicative of SRMA in young dogs. The rapid resolution of clinical signs with immunosuppressive steroid treatment is a very common feature of the acute form of the disease [1,3,13]. The chronic form frequently presents as cervical or multifocal myelopathy [3,22] and may be observed either following relapses of the acute form or if the course of treatment is inadequate [1,13]. The CSF analysis in chronic cases typically reveals mixed or mononuclear pleocytosis, with a normal or mildly elevated protein concentration [1,2,13].

As there are no clinical–pathological tests for its definitive identification, the suspected diagnosis of SRMA is based on the signalment, history, clinical and neurological exam findings, CSF analysis, CT scan, or magnetic resonance imaging (MRI) [1,2].

SRMA is strongly suspected after the exclusion of other diseases and by the positive clinical response to steroid and/or other immunosuppressive treatment [1,2]. During the chronic stage, the meningeal inflammation is generally less severe compared to the acute form [1,4] but the persistent inflammation can lead to nerve root degeneration and irreversible alterations of the meningeal structure due to fibrosis and sometimes mineralisation [4,11]. Additionally, in the chronic cases, the vessel walls within the meninges may thicken significantly. This thickening, and a possible secondary haemorrhage from the rupturing of the meningeal vessels, can cause spinal cord compression, ataxia, and paresis [4,11,13].

Spinal cord haemorrhage is categorised as either traumatic or non-traumatic [23]. Non-traumatic haemorrhage can result from conditions such as coagulopathies (*Angiostrongylus vasorum* infection, haemophilia, anticoagulant toxicity), neoplasia (hemangiosarcoma, granular cell tumour), vascular malformations, radiotherapy, or SRMA [23]. A study on 23 dogs with non-traumatic haemorrhagic myelopathy identified an underlying cause in about two-thirds of cases, with SRMA diagnosed in 3 dogs (13%) [24]. Although SRMA is recognised as a potential cause of haemorrhagic myelopathy, few case reports exist [25,26,27,28]. Reported haemorrhagic lesions have been localised in the epidural space [26], intradural–extramedullary space [27,28], or intramedullary region [25]. Haematomas, either extradural or intradural, are considered an emergency in human patients, with surgery being the gold standard treatment, with a success rate of approximately 40% of patients having a complete recovery of neurological deficits after surgery [29]. Magnetic resonance imaging is considered the imaging technique of choice when spinal cord haemorrhage is suspected [25,26,30]. The aim of our study is to highlight haemorrhagic myelopathy as an uncommon manifestation of the acute form of steroid-responsive meningitis-arteritis (SRMA), describing the signalment, clinical and neurological presentation, clinic-pathological results, CT or MRI findings, treatment, and outcome in dogs with haemorrhagic myelopathy secondary to SRMA.

## 2. Materials and Methods

### Study Designs, Inclusion, Exclusion Criteria, Medical Record Search, and Data Extraction

This retrospective, multicentre study reviewed the medical records of dogs diagnosed with haemorrhagic myelopathy secondary to steroid-responsive meningitis-arteritis (SRMA). Data were collected between January 2017 and June 2024 from five UK referral hospitals led by board-certified neurologists. Permission for publication was obtained by a signed consent for potential future research by the animal owners. Between October 2023 and June 2024, all five centres were contacted by email and agreed to participate. At least one member from each centre performed an independent electronic search of their medical record databases, covering cases seen between January 2017 and June 2024. The following keywords were used to identify eligible cases: “SRMA”, “steroid-responsive meningitis-arteritis”, “haemorrhagic myelopathy”, “paresis”, “paralysis”, and “plegia”. A total of twenty-two cases were identified across the centres. Cases were included based on specific criteria. A presumptive diagnosis of SRMA was required, based a combination of signalment, clinical examination findings (e.g., cervical or diffuse spinal pain), systemic inflammatory markers, cerebrospinal fluid (CSF) pleocytosis, the diagnostic imaging findings (CT or MRI), and, where available, a positive response to glucocorticoid or immunosuppressive treatment [31,32].

Pleocytosis was defined as a CSF total nucleated cell count (TNCC) greater than 5 cells/μL, characterised by a predominance of neutrophils or a mixed neutrophilic/monocytic profile, without cytological evidence of infectious agents [31,32]. In selected cases, further CSF analysis included polymerase chain reaction (PCR) testing for Toxoplasma gondii, Neospora caninum, and Canine distemper virus, as well as bacterial and fungal cultures.

Systemic inflammation was defined by the presence of at least one of the following: an elevated C-reactive protein (CRP) concentration (>10 mg/L), pyrexia (temperature > 39.0 °C) [33], or documented peripheral neutrophilia [32]. Only dogs that underwent complete general physical and neurological examinations, performed either by a board-certified neurologist or a European College of Veterinary Neurology (ECVN) resident under direct diplomate supervision, were included. The diagnosis of haemorrhagic myelopathy was based on MRI scan or surgical exploration or confirmed with post-mortem and histopathological examination. To rule out alternative causes of haemorrhagic myelopathy, particularly bleeding diatheses, additional diagnostic tests were performed at the attending clinician’s discretion. These included coagulation profiles (prothrombin time, activated partial thromboplastin time, fibrinogen levels, and D-dimers), platelet count, buccal mucosal bleeding time (BMBT), and *Angiostrongylus vasorum* antigen testing (Angio Detect Test, IDEXX Laboratories, Westbrook, ME, USA) [33]. Dogs with an incomplete medical record or when haemorrhagic myelopathy was resulting from traumatic injury, bleeding diathesis, iatrogenic trauma, or compressive or non-compressive intervertebral disc disease were excluded from the study [24]. Following the case identification, a systematic review of medical records was conducted to extract the following data: patient signalment; relevant history, including presenting complaint; onset of the clinical signs classified as peracute (<12 h), acute (12–72 h), subacute (3–14 days), or chronic (>14 days); course of the disease; and type of progression (progressive, waxing/waning, static, improving).

Findings of the general physical and neurological examinations: results of diagnostic procedures, including complete blood count (CBC), serum biochemistry, C-reactive protein levels (CRP), antigen testing for *A. vasorum* using an in-house assay (Angio Detect Test, IDEXX Laboratories, Westbrook, ME, USA), coagulation profile, buccal mucosal bleeding time (BMBT), and CSF analysis were recorded and evaluated.

Diagnostic imaging consisted of MRI, CT, and radiography reviewed by a board-certified neurologist or a board-certified radiologist, or both. MRI studies included the following: T2-weighted, T2-weighted (GRE), T1-weighted pre- and post-gadolinium. The lesions were classified as extradural, intradural–extramedullary, or intramedullary. Magnetic resonance imaging was performed using a 1.5 Tesla unit (Toshiba, Otawara, Japan; Siemens, Magnetom, Essenza), three low-field scanner 0.25 Tesla units (ESAOTE MR-Grande), and two 0.4 Tesla units (Hitachi, Aperto, Tokyo, Japan; Toshiba, Aperto Grande). Computed tomography scan was performed in selected cases with a 16-slice unit (Toshiba, Otawara, Japan). Finally, treatments, surgical interventions, complications, follow-up, prognosis, outcome, and, when available, the post-mortem and histopathological examination were recorded.

## 3. Results

### 3.1. Case Selection

**Figure 1:**
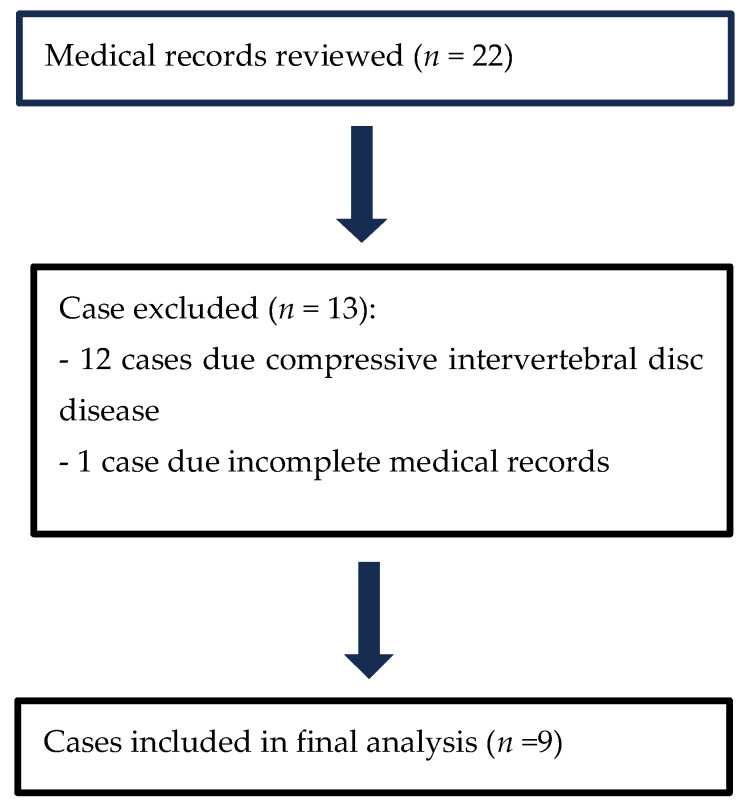
Summary of cases reviewed, excluded, and included.

Medical records of 22 dogs diagnosed with haemorrhagic myelopathy secondary to steroid-responsive meningitis-arteritis (SRMA) between October 2017 and February 2024 were reviewed. After applying the inclusion and exclusion criteria, nine cases were included in the final analysis. The case selection process is summarised in Figure 1.

### 3.2. Cases Included

The signalment (breed, sex, and age at presentation) and the body weight of each dog are listed in Table 1. The age ranged from 9 to 78 months (mean 19.11, SD ±20.94, median 12 months), and the mean body weight was 17.5 kg (SD ±7.07 kg, median 12.6 kg). The most common breed was the Whippet (*n* = 3), followed by the Beagle (*n* = 2).

### 3.3. History and Findings of Clinical and Neurological Examination

In our study population, the most common reported presenting complaint from the referring practice was cervical hyperesthesia and lethargy (*n* = 7, case 1, 2, 4, 5, 6, 7, 8) followed by pyrexia (*n* = 6, case 1, 2, 3, 4, 7, 9). Only four dogs (cases 1, 2, 3, and 6) were treated prior to referral. Treatment included intravenous fluid therapy, antibiotics, antipyretics, pain relief medications, and antiepileptic drugs. No consistent improvement on medication was observed in cases 1, 2, and 6, whereas case 3 showed a moderate resolution of clinical signs with paracetamol alone. None of the cases had received recent anthelmintic treatment. The onset of clinical signs was described as subacute in six dogs (cases 1, 2, 3, 4, 7 and 8) or acute (*n* = 3, case 5, 6 and 9). The median duration of signs before presentation was 4 days (range, <48 h to 9 days). The course of the disease was variable; eight dogs showed progressive neurological signs (cases 1, 2, 4, 5, 6, 7, 8 and 9), and one improved (case 3). At the referring hospital, clinical examination at presentation revealed pyrexia in cases 1, 2, 6, 7, and 9; bilateral scleral haemorrhage in two dogs (*n* = 2; cases 3 and 8); and unilateral scleral haemorrhage affecting the lateral and dorsolateral aspects of the left eye in case 4. The most common neurological sign at presentation was cervical hyperesthesia (*n* = 7, cases 1, 2, 4, 5, 6, 7, and 8). Motor deficits were variable, with ambulatory tetraparesis in one dog (case 6), tetraplegia with intact nociception in case 8, ambulatory paraparesis with delayed postural reactions in case 7, and two dogs presenting with paraplegia—one with absent nociception (case 2) and one with intact nociception (case 9). Additionally, one dog (case 1) developed paraplegia with absent deep pain perception three days after hospitalisation. Case 4 showed proprioceptive ataxia.

The neuroanatomical localisation varied within the group, being multifocal in four cases (cases 1, 2, 4, and 7). One case (case 9) was diagnosed with T3-L3 myelopathy. C1–C5 myelopathy was identified in one case (case 6), while case 8 had C6-T2 myelopathy. One dog (case 5) exhibited symptoms localised to the neck region. Only one dog (case 3) could not be localised due to a normal neurological examination. Table 2 presents the presenting complaints reported by the referring veterinary practice, while Table 3 summarises the physical and neurological findings from the referring hospitals.

### 3.4. Clinicopathologic Analyses

Haematology and serum biochemistry profiles were performed in all cases, C-reactive protein concentration (CRP) in six dogs (cases 1, 2, 3, 4, 5 and 8), and CSF analysis in seven cases (cases 1, 2, 3, 5, 6, 7, and 9). CSF analysis was performed with cisternal (*n* = 5, case 1, 5, 6, 7, 9) or lumbar tap (*n* = 1, case 3) or both (*n* = 1, case 2). Abnormal values in the haematology and biochemistry profile and results of the C-reactive protein concentration (CRP) are reported in Table 4. The CSF analysis results are shown in Table 5. *A. vasorum* antigen testing was negative in all the tested cases (*n* = 6, case 1, 3, 4, 7, 8, and 9), the prothrombin time (PT) and activated partial thromboplastin time (APTT) within normal limits in all the tested cases (*n* = 8, case 1, 2, 3, 4, 6, 7, 8, 9). Buccal mucosa bleeding time (BMBT) was assessed in two dogs and was 5 min in one dog (case 1), while platelet counts were normal in all cases (case 1, 2, 3, 4, 5, 6, 7, 8, 9). Tests of tertiary haemostasis were not assessed in any dog. Urinalysis revealed mild proteinuria (++) and haemoglobinuria (++++) in case 1. In case 8, a DNA test for haemophilia and ELISA test (SNAP 4Dx Plus Test Kit, IDEXX Laboratories, Westbrook, ME, USA) for A phagocytophilum, A platys, B burgdorferi, E canis, and E ewingii and antigen from D immitis were all negative. Toxoplasma gondii and Neospora caninum serology were also negative (*n* = 5, cases 1, 2, 5, 7 and 8). The coagulation parameters across the cases are summarised in Table 6.

### 3.5. Diagnostic Imaging Findings

**Figure 2:**
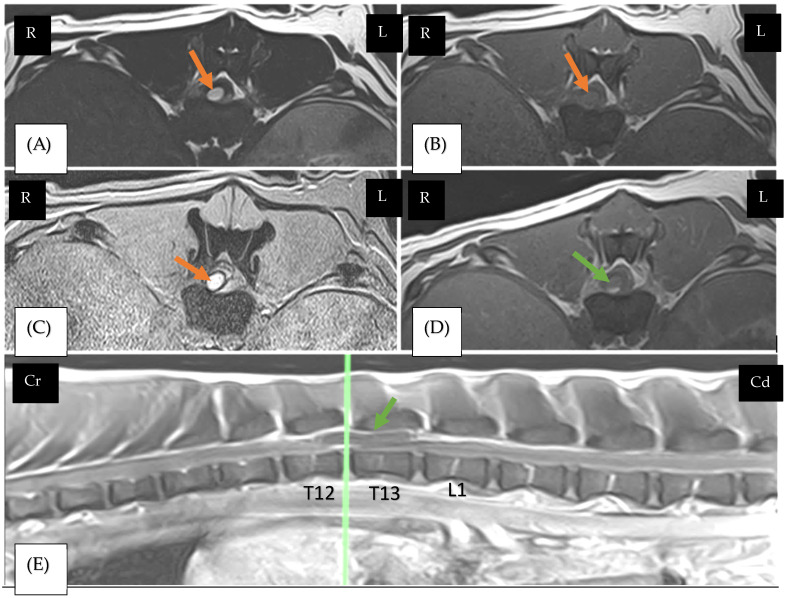
Crossbreed (case 1), male neutered, 9 M. transverse T2W image (**A**), transverse T1W image (**B**), and transverse T2* GRE image (**C**). Magnetic resonance shows intradural–extramedullary lesion compressing the spinal cord to the right at the level of T12-T13 (orange arrows). This corresponds to the site of the green line in the sagittal post-gadolinium-based contrast agent (GAD)-weighted image (**E**). Transverse T1W image of gadolinium-based contrast agent (GAD) (**D**), sagittal T1W image of gadolinium-based contrast agent (GAD) (**E**). Magnetic resonance shows a tubular well-defined lesion hypointense at level T12-L1 (green arrows) that shows a faint dependent contrast enhancement, indicating it is likely fluid-filled and possibly subject to active haemorrhage. It appears to be surrounded by the dura matter, indicating a mostly intra-dural component. The meninges show multifocal ill-defined enhancement. The peridural fat at this level is also partially attenuated and very heterogenous, indicating there is extra-dural involvement.

On MRI, T2-weighted (T2W) sequences were performed in all the dogs, revealing hyperintense lesions in six dogs (cases 1, 2, 6, 7, 8, and 9). T1-weighted (T1W) sequences, pre- and post-gadolinium, showed minimal heterogeneous enhancement across all cases, and T2*-weighted (gradient echo) sequences detected signal void intensities consistent with haemorrhage in eight dogs (cases 1, 2, 3, 4, 6, 7, 8, and 9), confirmed by histopathology in cases 2 and 8. The lesion was intramedullary in one dog (case 2), intradural–extramedullary in five dogs (cases 1 (Figure 2), 4, 6, 7, and 8), and extradural in two dogs (cases 3 and 5). The most frequent location for the haemorrhagic lesion was T3-L3 (*n* = 4, cases 1, 2, 7, and 9). Details of the MRI findings can be found in Table 7. A computed tomography (CT) scan was performed in two dogs. Case 1 underwent a CT scan of the head and spine on the day of presentation, which revealed no brain or spinal cord abnormalities. However, the CT scan was repeated 72 h later due to neurological deterioration, revealing a hyperattenuating lesion with dorsolateral compression of the spinal cord segments from T12 to L1. This lesion attenuated the epidural fat, occupying up to 80% of the vertebral canal in cross-section. There were multiple areas of heterogenous hypo-enhancement within the muscles of the thighs, suggesting myositis or muscle haemorrhages. In case 2, a CT scan without contrast media injection of the entire neuroaxis was performed under general anaesthesia on the day of presentation and was unremarkable. Thoracic radiographs were performed in case 4 and were unremarkable.

### 3.6. Diagnosis, Treatments, Outcomes, and Follow-Up

Considering the history, clinical signs, and results of the performed investigations, a presumptive diagnosis of steroid-responsive meningitis-arteritis (SRMA) was made in all cases. All the cases met the inclusion criteria; however, cases 4 and 8 were included despite the unavailability of CSF analysis. In case 8, CSF collection was attempted but was compromised by significant haemodilution, precluding analysis. In this case, the diagnosis of SRMA with haemorrhagic myelopathy was confirmed post-mortem. In case 4, SRMA was diagnosed based on the clinical presentation, elevated CRP, treatment response, and follow-up MRI findings, as CSF collection was complicated by haemodilution and was not repeated due to the risk of further haemorrhagic complications. The treatment was primarily focused on immunosuppressive therapy, with individual variations in response, clinical outcome, and treatment duration. Also, adjunct therapies and supportive care varied between cases, reflecting the different clinical presentations and the response to the initial treatment. Long-term immunosuppressive treatment was administered in five cases (case 3, 4, 5, 7, and 9), ranging from 11 weeks to seven months. Cases 3 and 5 received treatment for one month. Case 6 received treatment for two months but was lost to follow-up. In contrast, cases 1, 2, and 8 did not respond to immunosuppressive therapy and were euthanised due to a poor prognosis. Prednisolone was administered in six cases (cases 1, 2, 3, 4, 5, and 6), with doses ranging from 1 mg/kg once daily to 2 mg/kg twice daily, based on the treating clinician’s decision. Cytarabine, was administered in three cases (cases 6, 7, and 8), and was added upon those dogs relapsing or if the response to corticosteroids only was unsatisfactory. Mycophenolate was used as second-line immunosuppressant in case 1, and Cyclosporine after decompressive surgery in case 9. Supportive therapies included paracetamol as an antipyretic and analgesic. The five cases that responded well to early treatment (cases 3, 4, 5, 6, and 7) had a better outcome, improving over several weeks to months, and the immunosuppressive therapy was sustained and tapered appropriately, according to the clinical response. Case 4 showed a significant neurological improvement at the 11-week follow-up, and a nearly complete resolution of the intradural–extramedullary haemorrhage was demonstrated on a repeated MRI scan.

Relapses were observed in case 6 after steroid treatment was suspended, in some cases at variable times. A relapse occurred four months after the discontinuation of prednisolone, despite the initial improvement and clinical recovery.

The relapse manifested as a severe intradural extramedullary haemorrhage at the C3 level. After restarting immunosuppressive therapy with a combination of prednisone and cytarabine, the dog’s condition improved.

In contrast, some dogs had an initially more guarded prognosis, particularly those presenting with rapid neurological deterioration. Cases 1, 2, and 8 showed severe, progressive neurological deterioration, leading to euthanasia due to a poor prognosis for long-term quality of life. Case 1 deteriorated rapidly, developing paraplegia with absent nociception on day three of hospitalisation, due to an intradural–extramedullary haemorrhage. Despite aggressive medical management, including mycophenolate, tranexamic acid, and supportive care, the condition progressed irreversibly, resulting in euthanasia on day 13 after admission. Case 2 was persistently paraplegic, with loss of nociception five weeks after starting the immunosuppressive treatment with prednisolone and azathioprine, and unfortunately the prognosis remained poor, resulting in euthanasia. Similarly, case 8 presented with severe respiratory distress and progressive neurological deterioration despite the use of cytarabine and dexamethasone, leading to euthanasia. A notable difference in the response to treatment was observed in case 9 after a right-sided hemilaminectomy performed to address a compressive extradural haemorrhage at the T11–T12 level. The dog regained ambulation within two weeks and continued to improve over a seven-month period, eventually achieving a full neurological recovery. The treatments, including immunosuppressive dose rates, complications, follow-up, outcome, and prognosis, are listed in Table 8 and Table 9.

### 3.7. Post-Mortem Examination and Histopathology

**Figure 3:**
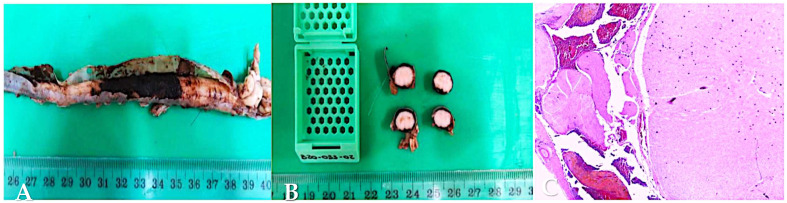
Beagle (case 2), female entire, 16 M. Macroscopic examination of spinal cord (**A**,**B**). Post-mortem image of the spinal cord illustrating intradural darkened appearance, compatible with haemorrhage. Microscopic examination of myelomeninges (**C**). Photomicrograph shows multifocal intradural haemorrhage with hemosiderin and hematoidin within the myelomeninges.

Post-mortem examination and histopathology were performed in two dogs (cases 2 and 8). In case 2, the entire spinal cord and brain was submitted for histopathologic examination. Macroscopic examination of the spinal cord revealed multifocal lesions with an intradural dark appearance, compatible with haemorrhage (Figure 3A,B). Multifocal intradural areas of haemorrhage admixed with hemosiderin and hematoidin in the spinal cord, within the myelomeninges, were microscopically evident (Figure 3C). The cerebrum and cerebellum did not show any abnormalities. The underlying spinal cord and nerve roots were histologically normal, despite the obvious compressive effects caused by the haemorrhage. No inflammatory changes were observed.

**Figure 4:**
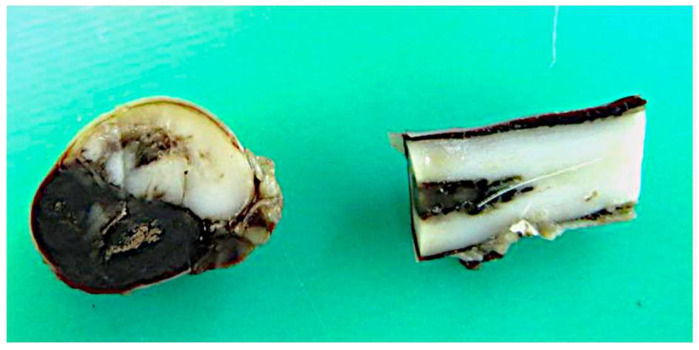
Beagle (case 8), female entire, 11 M. Macroscopic examination of spinal cord. Post-mortem image of the spinal cord shows a multifocal–coalescing subdural haemorrhage extending the length of the cervical vertebrae. On transverse section, half of the spinal cord is effaced by the haemorrhage.

In case 8, macroscopic examination of the fixed brain and spinal cord showed a multifocal–coalescing subdural haemorrhage extending the length of the cervical vertebrae. On transverse section, half of the spinal cord was effaced by the haemorrhage (Figure 4). Microscopic examination of the spinal cord revealed multifocal to coalescing, abundant extravasated red blood cells and neutrophils filling the subdural space and obliterating large portions of the spinal cord. The adjacent spinal cord showed a marked spheroid formation, multifocal areas of haemorrhage admixed with cellular debris (necrosis), and occasional red (ischaemic) neurones. Leptomeningeal vessel walls were often expanded by amorphous eosinophilic deposits (fibrinoid necrosis) and infiltrated by neutrophils, lymphocytes, and plasma cells showing signs of karyorrhexis (leukocytoclastic vasculitis). The brain revealed multifocal extravasated red blood cells expanding the subarachnoid space. Pale eosinophilic fluid expanded the perivascular spaces, with perivascular enlarged, foamy macrophages and glial activation (acute vasogenic oedema).

## 4. Discussion

Our study provides a detailed overview of the clinical presentation, magnetic resonance imaging findings, treatments, and outcomes of haemorrhagic myelopathy secondary to SRMA in dogs. In this study, Whippets were the most affected breed, consistent with findings by West et al. (2023) [24]. Mayor et al. (2025) reported vascular complications secondary to SRMA in seven dogs, six of which were Golden Retrievers [31]. While most studies suggest that SRMA affects both sexes equally [6,7,9,10,11], one publication reported a higher prevalence in male dogs [12]. Interestingly, our study contrasts with this finding, as seven out of nine cases were female. In this retrospective study, the median age at presentation was 12 months, with eight of nine dogs under 18 months of age [5,9,10]. One dog was 78 months old, and to our knowledge, this is the only reported case of haemorrhagic myelopathy secondary to SRMA at this age. Pyrexia was noted in six out of nine cases before referral and in five out of nine cases at presentation at the referring hospital. It was the most common clinical sign in SRMA cases in two large retrospective studies, reported in 81% of 100 dogs [34] and 82.6% of 350 dogs in another study [19]. In the present study, cervical hyperesthesia was the most common neurological sign at presentation, occurring in seven out of nine cases, which is consistent with the literature [6,34]. To our knowledge, no previous reports have associated SRMA with a clinical presentation of scleral haemorrhage, as observed in our study.

Specifically, two dogs (cases 3 and 8) had a bilateral scleral haemorrhage, and one dog (case 4) had a scleral haemorrhage on the lateral and dorsolateral sides of the left eye. Scleral and subconjunctival haemorrhages are often attributed to trauma; however, systemic causes such as coagulation disorders, vasculitis, and hypertension should also be considered [35]. In our study, scleral haemorrhages were observed without any history of trauma, prompting consideration of systemic causes. Among potential systemic causes, *Angiostrongylus vasorum*, a metastrongyloid nematode, is known to cause systemic bleeding diathesis in dogs [36]. All three cases tested negative for *A. vasorum* using the Angio Detect antigen assay, which has a high sensitivity (97.1%) and specificity (98.9%) [37], and none had received anthelmintic treatment prior to referral, eliminating potential confounding factors.

Additionally, all three dogs had normal platelet count, prothrombin time (PT), and activated partial thromboplastin time (aPTT) results, ruling out thrombocytopenia and suggesting no major abnormalities in the tested parameters of secondary haemostasis. One dog (case 8), with severe clinical signs, underwent additional testing for haemophilia and other infectious agents, including *A. phagocytophilum*, *A. platys*, *B. burgdorferi*, *E. canis*, and *D. immitis*, all of which yielded negative results. Also, in the latter case, post-mortem examination revealed extensive haemorrhagic and inflammatory CNS changes, suggesting SRMA. In contrast, the two other dogs (cases 3 and 4) improved with immunosuppressive therapy (prednisolone at 1 mg/kg PO q12h and 2.2 mg/kg PO q24h, respectively), showing a full resolution of clinical signs, including scleral haemorrhages, without relapse. However, it is important to note that a complete coagulation profile was not performed in all cases. Specifically, BMBT was assessed in two cases, while tests evaluating tertiary haemostasis (e.g., thromboelastography, fibrinogen levels, and D-dimers) were not conducted in any of the nine cases [33]. Given the retrospective nature of this study, certain diagnostic limitations are inherent. While secondary coagulopathies were effectively ruled out in eight of the nine cases (cases 1, 2, 3, 4, 6, 7, 8, and 9), the possibility of primary or tertiary coagulopathies cannot be dismissed. Future studies would benefit from a more comprehensive coagulation assessment to better elucidate the potential role of coagulation disorders in SRMA cases. It is possible that some dogs exhibited some degree of coagulopathy, most probably secondary to arteritis, which could explain the conjunctival bleeding.

Laboratory diagnostic tests and cerebrospinal fluid (CSF) analysis remain essential components in the diagnosis of SRMA [6]. In systemic diseases, one commonly observed laboratory alteration is leukocytosis with a left shift, indicating heightened white blood cell production [11]. A primary biomarker for systemic inflammation in dogs, which is also helpful in the diagnosis and management of SRMA, is C-reactive protein (CRP) [10,38]. However, CRP is rather a non-specific biomarker, and other inflammatory causes must be ruled out during its diagnostic consideration [6]. A complete blood count and serum biochemistry were performed in all nine cases, revealing leukocytosis, neutrophilia, and monocytosis in seven out of nine cases. CRP was measured in six out of nine dogs and was elevated in all tested dogs. In four cases, CRP was not repeated after starting immunosuppression treatment, because three dogs were euthanised, and one dog was lost to follow-up. CRP was repeated in two cases.

In both cases, one month after starting immunosuppression drugs, the CRP was found to be normal, with both dogs showing an improvement in clinical signs. CSF analysis is crucial for diagnosing SRMA [6]. To enhance its diagnostic accuracy and reduce false-negative results, CSF samples should be taken from both the cistern magna and the lumbar subarachnoid space. This dual approach, particularly effective in older dogs, significantly improves the detection of neutrophilic pleocytosis, as showed in a study of 111 dogs [8]. CSF analysis was performed in seven out of nine dogs, showing moderate to marked neutrophilic pleocytosis in all the tested dogs. Elevated erythrocyte counts and protein concentrations were observed in five out of seven dogs. CSF sampling was performed from both the cisterna magna and the lumbar subarachnoid space in only one instance (case 2), while the remaining cases involved single-site sampling. Nevertheless, this did not impact the diagnostic accuracy, as the findings were consistent with SRMA. A limitation of this study is the lack of immunoglobulin A (IgA) measurement in both serum and CSF to further support the diagnosis of SRMA. Increased IgA concentrations have been identified as a valuable biomarker for SRMA, with a diagnostic sensitivity of 91% and specificity of 78% when paired serum and CSF IgA measurements are applied [38,39]. IgA remains elevated even after treatment, highlighting its role in the disease process rather than solely as an acute-phase marker [38,39]. However, due to the retrospective nature of this study, these measurements were not available.

In one dog (case 8), CSF collection from the cisterna magna was attempted, but the sample was diluted by nearly pure blood and was not submitted for analysis. Due to the absence of CSF analysis, the diagnosis of SRMA associated with haemorrhagic myelopathy was made by necropsy. In another case (case 4), a diagnosis of SRMA was considered based on the patient’s signalment, history, clinical presentation, increased CRP concentration, response to immunosuppressive therapy, and follow-up MRI findings. Although CSF analysis was attempted, it was unsuccessful due to technical challenges, and further attempts were not pursued due to concerns regarding the risk of exacerbating haemorrhagic complications. Similar to previous reports, where the diagnosis of SRMA was based on clinical signs and increased CRP without CSF analysis [24], the diagnosis in this case was supported by compatible clinical features and a positive response to treatment. Additionally, a follow-up MRI conducted 11 weeks after the initial presentation showed a complete resolution of the changes in the subarachnoid space at C1–C3, without displacement or compression of the spinal cord.

While MRI is not always essential for diagnosis, it is often utilised in cases with atypical clinical presentations or due to institutional preferences [2,40,41]. MRI findings in SRMA are less frequently described, but, when observed, abnormalities are reported in 74% to 98.6% of cases [2,42]. These findings often include contrast enhancement in areas such as the meninges, articular facet synovium, paravertebral muscles, and cervical spinal cord parenchyma [2,42]. Among these findings, haemorrhagic myelopathy is rare, occurring in 9.4% [32]–13% [24] of cases, and it has been documented in a few case reports [6,25,26,27,28], with haemorrhages found in epidural space [26], intradural–extramedullary [27,28], or intramedullary [25]. Additionally, a recent study reported vascular complications in 21.2% of SRMA cases, including spinal and intracranial haemorrhages [31]. In our study, the most common haemorrhage site was the T3-L3 region, contrasting with previous reports that predominantly describe cervical involvement [6,26,27]. The neuroanatomical localisation matched the site of haemorrhage in all cases except for case 3, where the neurological examination was unremarkable despite an extramedullary haemorrhage at level C7-T1. We also noted that the haemorrhages were primarily right-sided. Few studies have shown that spinal arteries are more frequently located on the right side, with a statistically significant difference in the number of spinal arteries between the right and left sides, particularly in the cervical and thoracic regions [43,44]. This finding may explain why most haemorrhages were primarily located on the right side. The most common type of haemorrhage detected by MRI was intradural–extramedullary, observed in five out of nine cases. This type of haemorrhage affected the cervical region more. An intramedullary haemorrhage was detected in one dog, while extradural haemorrhages were found in three dogs. Neurological signs progressed during hospitalisation in five out of nine cases. One dog was ambulatory paraparetic (case 7), one was paraplegic with intact nociception (case 9), one was tetraplegic leading to severe respiratory distress (case 8), and two were paraplegic without deep pain perception (cases 1 and 2). Of these five cases with progressive neurological signs, three had intradural–extramedullary haemorrhages, one had a intramedullary haemorrhage, and one had an extradural haemorrhage. Therefore, the histopathological characteristics of SRMA, such as fibrinoid arteritis and systemic vasculitis, underscore the systemic nature of the disease [1]. Systemic vasculitis associated with SRMA often affects small to medium-sized arteries and can manifest in multiple regions, including the cervical spinal meninges and the heart [22,24]. A necroscopy was performed in cases 2 and 8, which confirmed spinal cord haemorrhages similar to previously described patterns of vascular involvement. Additionally, case 1 exhibited splenic infarcts and muscle haemorrhages, as revealed by CT imaging, further illustrating the systemic nature of the disease.

In humans, managing extradural and intradural haematomas that cause spinal cord compression is considered an emergency, with surgery being the gold standard treatment [29]. Surgical intervention results in complete recovery of neurological deficits in about 40% of cases [29]. Recent studies in veterinary literature have highlighted the potential benefits of surgical decompression in cases of spontaneous, non-traumatic haemorrhagic myelopathy in dogs [45]. This case series involving six dogs demonstrated that surgical decompression of extraparenchymal haematomas yielded favourable outcomes in the majority of cases (83%, or five out of six dogs) [45]. In a case report of SRMA, the treatment plan included a right-sided partial dorsal laminectomy at the level of C6, where the extradural haemorrhage was removed, followed by a course of immunosuppressive therapy using cytosine arabinoside. This choice was driven by concerns about the slow healing process often associated with conventional steroidal immunosuppressive drugs, such as prednisolone. This combined treatment proved successful, and notably, the dog did not develop any postoperative complications [26]. In another case, a right-sided hemilaminectomy at L2–L3 was performed to remove an intradural–extramedullary haemorrhage via a durotomy. Initially, the dog was treated with an anti-inflammatory dose of prednisolone, later increased to an immunosuppressive dose [28].

Interestingly, in our study, only one case (case 9) underwent surgical treatment and had a good outcome. A critical aspect of case 1 was the decision-making process regarding treatment approaches. Opting for medical management over surgery was influenced by factors including the immune-mediated nature of SRMA and potential bleeding risks, highlighted by a prolonged buccal mucosa bleeding time (BMBT). It is crucial to note that BMBT has its limitations and might not fully predict the risk of surgical bleeding [46,47]. In case 2, surgical treatment was not an option due to the owner’s choice for medical treatment. The literature indicates rapid clinical improvement with immunosuppressive doses of prednisolone in the acute cases of canine SRMA [1]. A variety of immunosuppressive medications have also been described to treat canine SRMA [1,2,48]. In this report, five dogs exhibited excellent outcomes following medical treatment. Of these five cases, three were treated exclusively with prednisolone. The remaining two cases received a combination therapy that included either prednisolone or dexamethasone, alongside cytarabine. This combination approach proved highly effective, contributing to the overall positive results observed in all five dogs. Breed differences are described for the clinical outcomes. According to Behr and Cauzinille (2006) [49], the prognosis for Boxers is much better than for Beagles and Bernese Mountain Dogs [47,50]. In our study, two of the three dogs that were euthanised were Beagles.

A small simple size, retrospective design, a variability in treatment protocols, and a lack of standardised MRI sequences were the limitations of this study. Despite these limitations, this study significantly enhances our understanding of the clinical presentation, MRI findings, and outcomes of haemorrhagic myelopathy secondary to SRMA in dogs. It emphasises the importance of a thorough diagnostic approach, combining medical history, physical and neurological examinations, clinical pathology, MRI, and CSF analysis to achieve a presumptive diagnosis.

## 5. Conclusions

This study highlights haemorrhagic myelopathy as a rare and severe complication of SRMA, underscoring the importance of recognising atypical clinical manifestations, particularly in its acute form. MRI findings predominantly localised haemorrhagic lesions to the thoracolumbar (T3-L3) region, with intradural–extramedullary haemorrhages being the most frequently observed. Immunosuppressive therapy with prednisolone, either alone or in combination with cytarabine, proved effective in most cases, while surgical intervention showed promise in managing a case of compressive extradural haemorrhage. Neurological deficits are more commonly associated with chronic SRMA, whereas acute cases typically present with cervical hyperesthesia and pyrexia. This clinical distinction may contribute to the underdiagnosis of SRMA in dogs presenting with vascular complications. Our findings highlight the need to consider SRMA as a differential diagnosis in dogs with spinal haemorrhage and neurological dysfunction. Future research should focus on refining treatment protocols for severe presentations and exploring the potential role of surgical intervention as a complementary approach in managing SRMA-related complications.

## Figures and Tables

**Table 1:** Breed, gender, age, and body weight.

Case No.	Case 1	Case 2	Case 3	Case 4	Case 5	Case 6	Case 7	Case 8	Case 9
Breed	Crossbreed	Beagle	Whippet	Border Collie	Boxer	Greyhound	Whippet	Beagle	Whippet
Sex	Male/ Neutered	Female/ Entire	Female/Entire	Female/Entire	Male/ Neutered	Female/ Neutered	Female/ Neutered	Female/Entire	Female/ Neutered
Age (months)	9	16	13	9	14	78	10	11	12
Weight (kg)	23.5	12.6	11.2	21.1	27.3	28.4	12.0	10.8	10.6

**Table 2:** Presenting complaints reported by the referring veterinary practice.

Case No.	Case 1	Case 2	Case 3	Case 4	Case 5	Case 6	Case 7	Case 8	Case 9
Presenting complains									
Pyrexia	X	X	X	X			X		X
Hyperesthesia	X	X		X	X	X	X	X	
Lethargy	X	X		X	X	X	X	X	
Inappetence	X								
Tremors		X							
Bilateral scleral haemorrhage			X						
Spastic paresis			X						
Seizures like episodes				X	X				
Monolateral scleral haemorrhage of the left eye				X					
Cervical hyperesthesia that progressed to tetraplegia								X	
Ambulatory tetraparesis						X			
Hemiparesis				X	X				

Abbreviations: X: signs present.

**Table 3:** Physical and neurological findings from referring hospitals.

Case No.	Case 1	Case 2	Case 3	Case 4	Case 5	Case 6	Case 7	Case 8	Case 9
Physical examination abnormalities									
Pyrexia	X	X				X	X		X
Bilateral scleral haemorrhages			X					X	
Monolateral scleral haemorrhage of the left eye				X					
Unremarkable					X				
Neurological signs									
Normal neurological examination			X						
Obtunded mentation				X					
Low head carriage				X			X		
Cervical hyperesthesia	X	X		X	X	X	X	X	
Ambulatory paraparetic with delayed postural reaction							X		
Paraplegic with intact nociception									X
Paraplegic with absent nociception	O	X							
Ambulatory tetraparetic						X			
Tetraplegic with intact nociception								X	
Proprioceptive ataxia				X					
Spinal reflexes reduced front limbs								X	
Anisocoria. The direct and indirect PLR was normal in the right eye				X					
Neuroanatomical localisation	(-)	(-)	+	(-)	Neck region	C1–C5 spinal cord segments	(-)	C6-T2 spinal cord segments	T3-L3 spinal cord segments

Abbreviations: X: signs present, PLR: pupilar light reflex, O: sign observed three days after hospitalisation. Multifocal: (-), +: = impossible to neurolocalise due to a normal neurological examination.

**Table 4:** Complete blood count, biochemistry, and C-reactive protein concentration abnormal results tests.

Case No.	Case 1	Case 2	Case 3	Case 4	Case 5	Case 6	Case 7	Case 8	Case 9
Neutrophil	25.5 × 10^9^/L; (RI 2.9–13.6 × 10^9^/L)	24.8 × 10^9^/; (RI 3.0–11.5 × 10^9^/L)	19.09 × 10^9^/L; (RI 3.0–12.0 × 10^9^/L)	12.51 × 10^9^/L; (RI 2.95–11.64 × 10^9^/L)	12.92 × 10^9^/L; (RI 2.95–11.64 × 10^9^/L)	(-)	12.14 × 10^9^/L; (RI 3.0–11.5)	19.32 × 10^9^/L; (RI 3.0–11.5)	(-)
Monocyte	5.4 × 10^9^/L; (RI 0.0 1.3 × 10^9^/L)	2.4 × 10^9^/L; (RI 0.0–1.3 × 10^9^/L)	1.64 × 10^9^/L; (RI 0.2–1.5 × 10^9^/L)	1.45 × 10^9^/L; (RI 0.16–1.12 × 10^9^/L)	1.55 × 10^9^/L; (RI 0.16–1.12 × 10^9^/L)	(-)	2.34 × 10^9^/L; (RI 0.2–1.4)	(-)	2.34 × 10^9^/L; (RI 0.2–1.4)
Eosinophil	(-)	(-)	(-)	(-)	(-)	0.02 × 10^9^/L; (RI 0.06–1.23 × 10^9^/L)	(-)	(-)	(-)
CK	1061 U/L; (RI 0–350 U/L)	(-)	(-)	(-)	(-)	(-)	697 IU/L; (RI 0–190)	(-)	(-)
ALT	(-)	170 U/L; (RI 13–78 U/L)	(-)	(-)	(-)	(-)	(-)	(-)	(-)
ALP	(-)	179 U/L; (RI 12–83 U/L)	(-)	(-)	(-)	(-)	224 IU/L; (RI 14–105)	444 IU/L; (14–105)	233 IU/L; (RI 14–105)
CRP	214 mg/L; (RI < 10.0 mg/L)	174.34 mg/L; (RI < 10.0 mg/L)	>82.8 mg/L; (RI < 10.0 mg/L)	>100 mg/L; (RI < 10.0 mg/L)	>100 mg/L; (RI < 10.0 mg/L)	*	*	336 mg/L; (RI < 10.0 mg/L)	*

Abbreviations: RI: reference interval, CRP: C-reactive protein concentration, (-): normal level, *: it was not performed, CK: creatinine kinase activity, ALT: alanine transaminase, ALP: alkaline phosphatase.

**Table 5:** CSF analysis results tests.

Case No.	Case 1	Case 2	Case 3	Case 4	Case 5	Case 6	Case 7	Case 8	Case 9
Location CSF tap									
Cisterna magna	X				X	X	X		X
Lumbar			X						
Both sites		X							
Unsuccessful				X				X	
Neutrophilic pleocytosis (RI: cells/μL)									
Marked									4896
Moderate	455	C: 262, L: 539	475		455	23	20		
RBC count (RI: cells/μL)									
Elevated	39,200	(^)	4056		3920				21,024
Protein concentration (RI: Cisterna < 30 mg/dL, Lumbar < 45 mg/dL)									
Elevated	115	C: 73.8 L: 70.6	191.95		115				214
CK (RI:0–40 IU/L)									
Elevated	314				324				
Bacterial culture									
Negative	X	X	(*)		X	(*)	X		(*)
PCR Toxoplasma gondii/Neospora caninum									
Negative	X	X	(*)		X	(*)	X		(*)
PCR distemper									
Negative	(*)	(*)	(*)		(*)	(*)	X		(*)

Abbreviations: RI: reference interval; CK: creatine kinase, C: cisterna magna; L: lumbar, (*): it was not performed, X: present. It was not reported: (^).

**Table 6:** Coagulation parameters results.

Case No.	Case 1	Case 2	Case 3	Case 4	Case 5	Case 6	Case 7	Case 8	Case 9	RI
PT	(-)	(-)	(-)	(-)	*	(-)	(-)	(-)	(-)	12.0–17.0 s
APTT	(-)	(-)	(-)	(-)	*	(-)	(-)	(-)	(-)	96–116 s
BMBT	5 min	*	*	*	*	*	*	(-)	*	<4 min

Abbreviations: RI: reference interval, PT: prothrombin time, APTT: activated partial thromboplastin time, BMBT: buccal mucosa bleeding time, (-): normal level, *: it was not performed.

**Table 7:** Summary of localisation, type of lesion, and T1-weighted, T1-weighted post-gadolinium, T2-weighted, and T2*-weighted (gradient eco) signal intensities.

Case No.	Localisation of Haemorrhage	Haemorrhage Type	Side of Haemorrhage	T1W/T1W Post-GAD	T2W	T2*W
1	T12-L1	Intradural– Extramedullary	Right-sided	Hypointense/poor enhancement	Hyperintense	Hypointense
2	T4–T5	Intramedullary	Diffuse	Hyperintense/no enhancement	Hyperintense	Hypointense
3	C7-T1	Extramedullary	Ventral and left-sided	Hyperintense/poor enhancement	Hypointense to isointense	Hypointense
4	C1–C3	Intradural– Extramedullary	Ventral	Hyperintense to isointense/poor enhancement	Hyperintense to isointense	Hypointense to isointense
5	T1–T2	Extramedullary	Right-sided	Hypointense/poor enhancement	Hyperintense to isointense	(*)
6	C3	Intradural– Extramedullary	Left-sided	Hypointense/poor enhancement	Hyperintense	Hypointense
7	T9-L3	Intradural– Extramedullary	Dorsal and right-sided	Hyperintense/poor enhancement	Hyperintense	Hypointense
8	C5	Intradural– Extramedullary	Ventral and left-sided	Hyperintense/poor enhancement	Hyperintense	Shows peripheral susceptibility artefacts
9	T11-L3	Extradural	Dorsal and right-sided	Hyperintense/poor enhancement	Hyperintense	Diffuse signal void surrounding the cord

Abbreviations: T1W: T1-weighted, T1W post-GAD: T1-weighted post-gadolinium, T2W: T2-weighted, T2*W: weighted (gradient eco), (*): it was not performed.

**Table 8:** Summary of treatment during hospitalisation and surgery.

Case No.	Case 1	Case 2	Case 3	Case 4	Case 5	Case 6	Case 7	Case 8	Case 9
Immunosuppressive treatments									
Prednisolone	1 mg/kg PO q24h from D 1	1.25 mg/kg PO q12h from D 2	1 mg/kg PO q12h for 1 M	2.2 mg/kg PO q24h for 1 M	2.2 mg/kg PO q24h for 1 M	2 mg/kg PO q12 started 4 M after discontinuation			
Dexamethasone		0.2 mg/kg IV from D 1 (case 2)					0.166 mg/kg PO q24 for 6 M	0.166 mg/kg PO q24h for 5 D	0.2 mg/kg PO q24h for 5 M
Cytarabine						200 mg/m^2^ CRI every 3 W	200 mg/m^2^ SC single dose	200 mg/m^2^ CRI single dose	
Mycophenolate	12 mg/kg PO q24h from D 4								
Ciclosporine									5 mg/kg PO q12h for 7 M, tapered after 5 M
Azathioprine		2 mg/kg PO q24h from D 2							
Supportive therapy									
Paracetamol	15 mg/kg IV q8h from D 1		10 mg/kg PO q8h for 1 M	15 mg/kg PO q8h for 2 W	15 mg/kg PO q8h for 1 M				
Amoxicillin	12 mg/kg PO q8h from D 3								
Tranexamic acid	25 mg/kg PO q8h from D 4 (case 1)								
Levetiracetam				30 mg/kg PO q8h ongoing					
Surgery									Right-sided haemilaminectomy at T11–T12 on D 1

Abbreviations: D: day, W: week, M: month.

**Table 9:** Summary of complications, follow up, outcome, and prognosis.

Case No.	Case 1	Case 2	Case 3	Case 4	Case 5	Case 6	Case 7	Case 8	Case 9
Complications									
Paraplegia with absent nociception	Developed on D 3	Persistent on W 5							
Paraplegia with nociception									Initially, but regained ambulation by D 14
Severe hypoventilation								Developed on D 5	
Relapse						4 M after prednisolone discontinuation			
Mild ataxia				Present still on D 30					
Follow-up Findings									
Complete recovery			In 1 M	In 4 M	In 4 M	In 1M	In 6 M		Regained ambulation in 2 W
No recovery	X	X						X	
Repeated MRI				At W 11 showed resolution of lesions					
Outcome									
Euthanised	X	X						X	
Full recovery			X	X	X	X	X		X
Prognosis									
Poor	X	X						X	
Good			X	X	X	X	X		X

Abbreviations: D: day, W: week, M: month, X: present.

## Data Availability

The data that support the findings of this research are available from the corresponding author upon reasonable request.
